# Calcium Intake and Body Composition in African-American Children and Adolescents at Risk for Overweight and Obesity

**DOI:** 10.3390/nu2090950

**Published:** 2010-09-10

**Authors:** Frances A. Tylavsky, Patricia A. Cowan, Sarah Terrell, Merschon Hutson, Pedro Velasquez-Mieyer

**Affiliations:** 1 Department of Preventive Medicine, University of Tennessee Health Science Center, 600 Jefferson Avenue, Suite 300, Memphis, TN 38112, USA; 2 College of Nursing, University of Tennessee Health Science Center, 920 Madison Avenue, Suite 507N, Memphis, TN 38103, USA; Email: pcowan@uthsc.edu; 3 Department of Preventive Medicine, University of Tennessee Health Science Center, 600 Jefferson Avenue, Suite 300, Memphis, TN 38112, USA; Email: shencyk@uthsc.edu; 4 General Clinical Research Center, University of Tennessee Health Science Center, 8 East Methodist University Hospital, Memphis, TN 38104, USA; Email: mhutson4@uthsc.edu; 5 Lifestyle Diabetes and Obesity Center, University of Tennessee Health Science Center, 1068 Cresthaven, Suite 300, Memphis, TN 38119, USA; Email: velasquez493@gmail.com

**Keywords:** dietary calcium, overweight, adolescents, hypertension, dyslipidemia, glucose metabolism

## Abstract

This study examined the role of calcium intake on body composition in 186 African-American adolescents at risk for overweight and obesity. The average weight of 89.8 kg ± 23.6 (SD) had a mean BMI z score of 2.2. Females with a calcium intake of <314 mg/day had higher percent fat mass compared to those with the highest calcium intakes that were ≥634 mg/day. Compared to those with a low calcium intake (<365 mg/day), those with the highest calcium intake of >701 mg/day had higher intake of thiamin, folate, cobalamin, vitamin D, phosphorus, iron, zinc.

## 1. Introduction

The threat of obesity is greater than ever for US children and adolescents [1,2]. Almost 31.9% of children between age 2 and 19 years in the US are at risk for overweight, *i.e.*, >85th percentile for body mass index (BMI) for age and gender] and overweight (≥95th percentile for BMI for age and gender [3]. All indications are that the current generation of children will grow into the most obese generation of adults in US history [4]. Although obesity has affected all socio-demographic strata in the US in an epidemic fashion, minorities have a disproportionately higher risk for obesity [1,2,5]. African-African (AA) adolescents are almost twice as likely to be affected as Caucasians [2,6,7,8] and consume less than half of the recommended amount of dairy products and calcium on a daily basis [9]. 

Dietary calcium intake has been negatively related to body weight and/or body fat in studies of children and adolescents [10,11,12,13]. In Caucasian females, lower intake of calcium or of dairy products has been associated with higher body fat [10] and a lower variety of food selection. Higher calcium intake or dairy product consumption was associated with decreased iliac skinfold thickness but not body weight in a group of Caucasian-Asian-Pacific Islanders [11]. Lower dairy intake is often associated with lower nutrient density, which raises the question of whether the level of fat mass is attributed to low dairy intake or to an overall diet that is limited in key nutrients. Cardiometabolic syndrome is a cluster of metabolic dysfunctions including obesity, hypertension, low-dyslipidemia, proinflammatory state, and insulin resistance/glucose intolerance in children [14]. In both children and adults, it has been associated with increased risk of coronary heart disease and type 2 diabetes. Different components of metabolic syndrome are widely prevalent in children and adults with overweight or obesity. While lifestyle modification is recommended for management of the syndrome, the dietary pattern most beneficial for patients is yet to be ascertained. High dairy intake has been generally associated with reduced risk for components of metabolic syndrome including type 2 diabetes, dyslipidemia, obesity and hypertension in adults [15,16] but with no evidence in children.

However, results have been very inconsistent across studies, and few studies have examined the effect of dairy intake in children and adolescents [17]. Therefore, the primary objective of this paper was to examine the role of calcium intake on fat mass (FM), bone free lean mass (LM), bone mass (BMC) and BMI in AA children and adolescents classified as “at risk for overweight” or “overweight” between the ages of 11–18 years. As a secondary objective, we thought to evaluate the association between calcium intake and the presence of different components of metabolic syndrome, including dyslipidemia, hypertension, glucose abnormalities, and severity of overweight, and also to examine the associations between the levels of calcium intake and the intake of macronutrients in the diet for macronutrients, vitamins and minerals.

## 2. Experimental Section

### 2.1. Participants

The 186 participants enrolled in this study were AA males or females (11–18 yrs.) whose BMI was ≥85th percentile for age and gender on the Centers for Disease Control growth charts. The participants were a convenience sample that was recruited to examine subclinical cardiovascular and diabetes risk factors in overweight children/adolescents. Individuals were excluded if there were physical limitations affecting the ability to complete the exercise test; previous diagnosis of diabetes mellitus, hypertension, liver, kidney or heart disease; current medication therapy that affects body weight, glucose or lipid metabolism, including estrogen, contraceptives or anticoagulants; pregnancy; documented cardiac defect; cognitive impairment (more than 2 grades behind age-appropriate grade in school); or weight greater than 350 lbs (upper limit for equipment). The University of Tennessee’s Institutional Review Board approved the study protocol. Written consent was obtained from those participants 18 years of age. For those less than 18 years of age, written assent was obtained with written consent from the parents/legally authorized representative. 

### 2.2. Measurements

#### 2.2.1. Three-Day Food Diary

Participants and their legally authorized representatives were instructed on recording 3 days of food intake. One weekend and 2 week days were recorded. Emphasis was placed on estimating portion sizes and description of food and drink consumed. A registered dietitian reviewed the completed record with the participant for completeness using food models. NutriBase 2001 clinical nutritional analysis software program (CyberSoft, Inc.) was used to determine macro- and micronutrient intake. The percent of participants whose intake met the dietary recommended intake (DRI) level was determined for each nutrient [18,19,20,21,22], and the acceptable macronutrient distribution range (AMDR) was calculated for protein, carbohydrate and fat [22]. Of the participants enrolled, 162 completed 3 days, and 24 completed 2 days of records. The intake of the 3 or 2 days was averaged to reflect the nutrient intake of the participant. 

#### 2.2.2. Blood Pressure

The participant rested in a seated position for 15 minutes prior to the first blood pressure measurement. Trained personnel selected the appropriate size cuff based on upper arm circumference. Two blood pressure readings were taken 5 minutes apart and averaged to reflect the participant’s blood pressure. Individuals with systolic or diastolic blood pressure ≥95th percentile for age, gender, and height were considered hypertensive [23]. For those <12 years of age, pre-hypertension was defined as a systolic or diastolic BP elevation >90th and <95th percentiles for age, gender and height [23]. For those 12 years and older, prehypertension was defined as a blood pressure ≥120/80 mm Hg but <95th percentile for age, gender and height [24].

#### 2.2.3. Lipid Measurements

Fasting blood samples were obtained for a lipid profile. Total cholesterol, HDL-cholesterol, and triglyceride were measured with an automated analyzer (Roche Cobas-Mira) utilizing the manufacturer’s commercially available kits; LDL-cholesterol was calculated using the Friedewald formula by the University of Miami Diabetes Institute. For this study, lipoprotein values ≥95th percentile for total cholesterol, triglycerides, and LDL-cholesterol and HDL-cholesterol values ≤5th percentile were considered abnormal. Values between the 90th and 95th percentile for cholesterol, triglycerides, and LDL-cholesterol or between the 5th and 10th percentile for HDL-cholesterol were classified as borderline.

#### 2.2.4. Glucose Measurements

An oral glucose tolerance test (OGTT) was performed after an overnight fast. Participants consumed 1 gm dextrose/kg of body weight (Allegiance, MacGaw Park, IL) up to a maximum of 75 gm. Blood samples were obtained at 0, 15, 30, 60, 90, and 120 minutes. The 2007 ADA diagnostic guidelines were used to distinguish normal versus impaired glucose metabolism (IGM). Subjects with impaired fasting glucose (IFG), impaired glucose tolerance (IGT) or diabetes were considered with IGM. Subjects with fasting level of glucose between 100–125 mg/dL were IFG, if two-hour plasma glucose level between 140–200 mg/dL were considered IGT, and diabetes was defined as a fasting glucose ≥126 mg/dL or a two-hour postprandial glucose level of ≥200 mg/dL. Serum glucose and insulin (µU/mL) levels were measured by glucose oxidase [25] and standard double-antibody radioimmunoassay respectively (Linco Research; St. Louis, MO).

#### 2.2.5. Body Composition from Dual Energy X-ray Absorptiometry (DXA)

Measurement of total mass, bone free lean mass, fat mass, bone area, bone mineral content and bone density of the whole body was performed using the Hologic Discovery A (Bedford, MA) software version 8.3. A DXA-certified research nurse performed the DXA measurements using a standardized protocol. Those individuals who were too large to fit within the limits of the scan region were scanned with correct placement of the right arm and part of the left arm out of region of interest. Right arm values were substituted for the left arm values (N = 30). All DXA scans were reviewed for quality assurance by study investigators Dr. Velasquez-Mieyer or Dr. Tylavsky.

#### 2.2.6. Anthropometric Measurements

Weight was recorded in kilograms using a Tanita BWB-800s Digital Scale with minimal indoor clothing without shoes. Height was recorded in centimeters using a stadiometer. BMI was calculated as weight/height^2^. Relative BMI was calculated as BMI divided by BMI from the 50th percentile for age and gender of the CDC growth charts.

### 2.3. Statistical Analyses

All data were analyzed using JMP 7.0 (2008 SAS Institute, Cary, NC). Means and standard deviations were determined for all continuous variables. The categorical variables are reported as the percent of the total sample size. Body composition measurements were the dependent variables. Calcium intake was the independent variable. Analyses were performed using calcium as a continuous variable and as calcium percentile groups. Calcium groups were defined by percentile within the study population and for males and females separately. When females and males were combined, the lowest calcium group included those in the <25th percentile (<365 mg/day); the middle group as those who fell into the 25th–74th percentile (365–700 mg/day) and the highest group those ≥75th percentile (≥701 mg/day). For females, the lowest calcium group was <314 mg/day, the middle group was 315–633 mg/day and the highest group was ≥634 mg/day. For males, the lowest group was <441 mg/day, the middle group was 442–783 mg/day, and the highest group was ≥784 mg/day. Age and energy intake were included as covariates within gender groups to control for possible confounding between calcium intake and body composition by DXA or relative BMI. A p value less than 0.05 was considered to be a significant association between calcium intake and body composition by DXA or relative BMI. Sensitivity analyses were performed to determine if there was an effect of calcium quartile cut points on body composition within each gender group. Reporting of energy intake compared to energy requirements were used to estimate reporting errors. Differences in nutrient intake between gender and age groups were evaluated using a t-test with a Bonferonni adjustment for multiple comparisons, p < 0.05. As secondary analyses, nutrient intake was compared across the calcium percentile groups using analysis of covariance with nutrient intake as the dependent variable and calcium percentile group as the independent variable controlling for energy intake, misreporting error and age. 

## 3. Results and Discussion

### 3.1. Results

Our participants represented a convenience sample of AA children with a wide-range overweight (Table 1). The weight of the participants increased with age. The mean BMI z-score (2.2–2.5 for age and gender categories) represents those individuals who were in the upper range for body mass. Based on DXA results, this sample had a median percent of body fat of 42% and the values ranged from 16% to 58%. The prevalence for elevated blood pressure was 55.5%; 25.6% for impaired glucose metabolism, and 51.1% for dyslipidemia. Table 1 provides a breakdown of the prevalence for these disorders by age and gender groups. 

**Table 1:** Characteristics of the study population by gender and age.

	Female		Male	
	11–13.9 (n = 32)	14–18 (n = 80)	11–13.9 (n = 28)	14–18 (n = 46)
	Mean ± SD	Mean ± SD	Mean ± SD	Mean ± SD
Weight (kg)	85 ± 23	102 ± 22	87 ± 22	110 ± 21
Height (cm)	158 ± 6	162 ± 8	163 ± 11	170 ± 7
BMI (kg/m^2^)	34 ± 9	38 ± 8	33 ± 7	38 ± 7
BMI Z score	2.2 ± 0.5	2.2 ± 0.4	2.3 ± 0.4	2.5 ± 0.5
Relative BMI (%)	184 ± 51	189 ± 37	179 ± 37	185 ± 33
Whole Body Bone Mineral Content (kg)	2.1 ± 0.3	2.5 ± 0.3	2.1 ± 0.4	2.7 ± 0.4
Whole Body Bone Mineral Density (g/cm^2^)	1.02 ± 0.09	1.16 ± 0.08	1.00 ± 0.10	1.14 ± 0.11
Fat Mass (kg)	38 ± 16	47 ± 14	35 ± 15	44 ± 15
Fat Free Mass (kg)	48 ± 8	55 ± 9	51 ± 10	66 ± 9
Total Mass (Fat + Fat Free Mass) (kg)	86 ± 23	102 ± 22	87 ± 22	110 ± 21
Body Fat (%)	43 ± 7	45 ± 6	40 ± 9	39 ± 7
Systolic Hypertension (%)				
Normotensive	50.0	45.0	48.3	37.8
Prehypertension	18.8	22.5	20.7	26.7
Hypertension	31.3	32.5	27.6	35.6
Diastolic Hypertension (%)				
Normotensive	96.9	85.0	89.7	95.6
Prehypertension	3.1	10.0	3.4	4.4
Hypertension	0	5.0	6.9	0
Impaired Glucose Metabolism	31.3	20.3	22.2	33.3
Dyslipidemia	51.6	43.0	52.2	64.4

The participants reported an average energy intake of 1794 kilocalories with a median of 1746 kilocalories per day. The energy intake reported by our sample is 940 kcal lower than what would be expected using the equations to determine energy requirement (EER) for overweight males and females [21]. Both males and females reported lower intake of calories compared to the EER (−1374 for males and −653 for females, p < 0.001). The ratio of reported energy intake to EER was 0.68 ± 0.29. Table 2 provides a breakdown for nutrients consumed by our participants by age and gender groups.

**Table 2:** The breakdown for nutrients consumed by age and gender groups.

	Female	Male	Female	Male
	11–13 y (n = 32)	11–13 y (n = 28)	14–19 y (n = 80)	14–19 y (n = 46)
	Mean ± SD	Mean ± SD	Mean ± SD	Mean ± SD
Energy (kcal)	1775.6 ± 631.5	1902.6 ± 529.7	1688.7 ± 700.1	1926.4 ± 416.1
Protein (g)	68.1 ± 24.2	72.7 ± 21.8	63.4 ± 24.9	74.7 ± 22.3
Carbohydrate (g)	216.6 ± 78.9	230.0 ± 72.9	200.6 ± 99.3	234.5 ± 55.5
Fat (g)	71.9 ± 29.0	78.0 ± 23.8	71.2 ± 33.0	78.7 ± 19.9
Fiber, total dietary (g)	9.1 ± 3.5	9.8 ± 5.2	9.3 ± 5.0	10.0 ± 4.3
Riboflavin (mg)	1.0 ± 0.6	1.1 ± 0.5	0.9 ± 0.5	1.1 ± 0.6
Folate (mcg)	161.2 ± 112.2	174.4 ± 100.7	158.2 ± 107.0	181.5 ± 156.3
Vitamin B-12 (mcg)	5.1 ± 6.6	2.9 ± 2.9	2.9 ± 5.3	2.9 ± 3.8
Vit-A (mcg_RAE)	284.6 ± 417.5	181.4 ± 126.2	176.9 ± 153.8	223.2 ± 193.9
Vitamin D (IU)	65.3 ± 74.2	63.2 ± 49.4	41.5 ± 57.5	56.1 ± 71.4
Tocopherol, alpha (mg)	1.1 ± 0.9	1.5 ± 1.2	1.7 ± 2.0	1.5 ± 2.0
Vitamin K (mcg)	17.3 ± 33.6	20.3 ± 38.2	26.8 ± 45.4	15.3 ± 24.5
Vitamin C (mg)	46.8 ± 37.2	63.7 ± 50.4	52.9 ± 45.4	69.1 ± 51.6
Calcium (mg)	509.0 ± 254.8	671.8 ± 280.7	489.3 ± 252.3	613.9 ± 236.0
Magnesium (mg)	87.0 ± 50.4	112.9 ± 54.5	100.1 ± 69.9	94.8 ± 58.6
Phosphorus (mg)	572.0 ± 374.6	738.1 ± 389.4	552.1 ± 361.0	547.3 ± 365.9
Potassium (mg)	982.3 ± 446.3	1364.0 ± 643.0	1218.7 ± 833.1	1186.6 ± 615.7
Iron (mg)	11.0 ± 4.4	10.5 ± 3.1	10.4 ± 6.5	12.1 ± 4.6
Zinc (mg)	4.6 ± 3.3	5.4 ± 2.7	5.2 ± 4.0	5.0 ± 3.8

**Figure 1:**
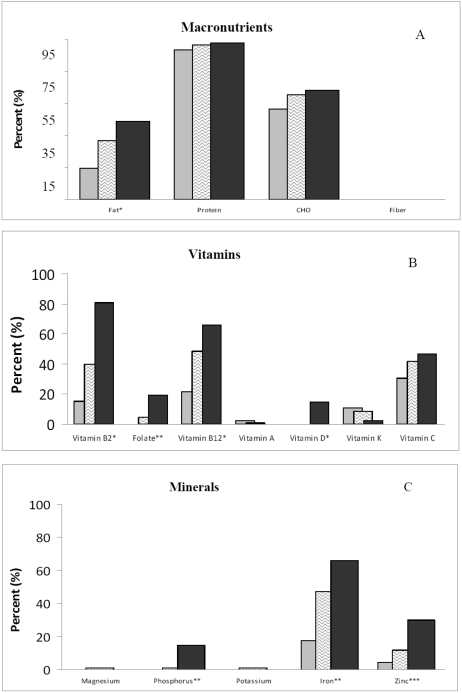
Percent of AA adolescents who met the (**A**) Acceptable Macronutrient Distribution Range (ADMR); the Recommended Dietary Intake of (**B**) selected vitamins; and (**C**) minerals [18,19,20,21,22] by calcium percentile intake groups [low (<365 mg/d), 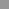; middle (365–700 mg/d), 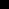; and high (>701 mg/d), 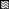]. Significance for analysis of variance across groups adjusted for energy and gender: *p < 0.01; **p < 0.001; ***p < 0.0001.

In our sample of AA children/adolescents, 96.2% met the AMDR for protein; 35.5% for fat, and 64% for carbohydrate. Less than half of the participants met the DRI for any other nutrient (Figure 1): calcium (1.1%); magnesium (4.3%); phosphorus (4.3%); potassium (0.5%); iron (44.6%); zinc (14.5%); folate (7.0%); riboflavin (44.1%); and vitamin B_12_ (46.2%); vitamin C (40.3%); vitamin D (3.8%); vitamin A (1.1%); or vitamin K (7.5%). None of the participants met the DRI for fiber or alpha tocopherol. Females reported less energy intake (1714 ± 57 *vs.* 1916 ± 70, p < 0.03) and lower average calcium intake (495 ± 24 *vs.* 639 ± 29, p < 0.0001) than the males did. 

**Figure 2:**
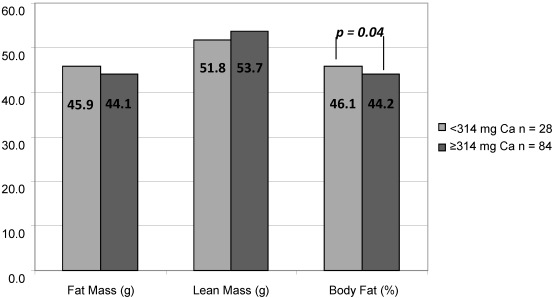
Body composition from dual energy X-ray absorptiometry for African-American females ages 11–18 in relation to calcium intake adjusted for energy intake (n = 112).

There was no association between calcium intake and FM, LM, BMC or relative BMI when the whole sample was examined, while adjusting for age and energy intake. In a gender-specific investigation, those females in the lowest calcium-intake group had higher % FM, than those in the middle and highest calcium-intake group while controlling for age and energy intake (Figure 2). There was no association with relative BMI. For males, there was no association between the lowest and highest quartile of calcium intake and any body composition measures. 

Nutrient intake was compared across calcium-intake groups, adjusting for age, gender and energy intake. As expected, those with the lower calcium intake had lower energy-adjusted intake of fiber, selected vitamins (riboflavin, folate, vitamin A, vitamin D) and mineral intake (magnesium, phosphorus, potassium, iron and zinc)(Table 3). Those in the highest calcium-intake group had a higher percentage of participants who met the ADMR for fat and met the DRI for most nutrients, with the exception of vitamins A and C and magnesium and potassium. 

**Table 3:** Dietary intake of participants (Mean and SD) by calcium-intake groups adjusted for energy, age and gender.

	Calcium-Intake Group		
	<365 mg/day (n = 46)	365–700 mg/day (n = 93)	≥701 mg/day (n = 48)
Protein (g)	64.0 ± 2.4	69.8 ± 1.6	70.3 ± 2.4
Fat (g)	76.7 ± 1.8	74.2 ± 1.2	72.1 ± 1.8
Carbohydrates (g)	215.6 ± 5.1	215.4 ± 3.4	221.2 ± 4.9
Lactose (g) ^3^	0.6 ± 0.7	1.8 ± 0.5	4.6 ± 0.7
Fiber, total dietary (g) ^1^	7.9 ± 0.6	9.9 ± 0.4	10.3 ± 0.6
Riboflavin (mg) ^2^	0.6 ± 0.1	0.9 ± 0.0	1.5 ± 0.1
Folate (µg) ^2^	102.1 ± 17.1	164.5 ± 11.4	239.3 ± 16.5
Vitamin B_12_ (µg)	2.1 ± 0.8	3.4 ± 0.5	4.2 ± 0.7
Vit-A (mcg_RAE) ^3^	132.3 ± 34.2	186.9 ± 22.9	324.4 ± 33.0
Vitamin D (IU) ^2^	7.9 ± 8.3	45.5 ± 5.5	108.3 ± 8.0
Vitamin K (mcg)	25.1 ± 6.0	20.2 ± 4.0	20.8 ± 5.7
Vitamin E (mg)	1.6 ± 0.3	1.5 ± 0.2	1.5 ± 0.3
Vitamin C (mg)	55.0 ± 7.1	59.7 ± 4.7	56.8 ± 6.8
Magnesium (mg) ^2^	66.0 ± 8.8	99.7 ± 5.9	130.3 ± 8.5
Phosphorus (mg) ^2^	378.7 ± 48.1	541.2 ± 32.2	867.9 ± 46.4
Potassium (mg) ^2^	872.8 ± 98.7	1171.7 ± 66.0	1565.9 ± 95.2
Iron (mg) ^2^	8.9 ± 0.8	11.2 ± 0.5	12.9 ± 0.7
Zinc (mg) ^2^	3.3 ± 0.5	4.9 ± 0.3	7.2 ± 0.5

^1^ p < 0.02; ^2^ p < 0.0001; ^3^ p < 0.001 for comparison across calcium group adjusted for age, gender and energy intake.

Those in the highest calcium-intake group had a higher percentage of participants who met the ADMR for fat and met the DRI for most nutrients with the exception of vitamins A and C and magnesium and potassium (Figure 1). When controlling for misreporting the results remained the same.

In our sample of adolescents, we found no association between calcium intake and dyslipidemia, impaired glucose metabolism, hypertension, severity of overweight or combined risk of having one of these. Similarly, the mean calcium intake was not different among subjects with and without dyslipidemia (549 ± 27 *vs.* 537 ± 27), IGM (570 ± 38 *vs.* 542 ± 22), or hypertension (557 ± 26 *vs.* 540 ± 29), nor was there difference comparing the least with more severe overweight (541 ± 24 *vs.* 570 ± 31).

### 3.2. Discussion

To our knowledge, this is the first paper that documents dietary intake and body composition of AA children and adolescents whose BMI is 111% above the 50th percentile for age and gender. The nutrient intake reflects a diet of foods low in nutrient density. Less than 10% of the sample met the DRI for calcium, magnesium, phosphorus, potassium, folate, fiber, and vitamins D, A and K. In this convenience sample of AA youth with extremely high body weight, calcium intake as a continuous variable was not related to % FM, FM, LM, or BMI for males or females. In females only, those in the lowest calcium percentile group (<314 mg/day) had higher % FM, compared to those in the highest calcium percentile group. There were no associations between diet and body composition in males.

High intake of dairy products has been shown to have beneficial effects on body weight in children and adults [26] in some but not all studies [13,27]. Studies show there is no effect of dairy products on change in weight or fat mass in young girls occurred during rapid growth [13]. In contrast an observational study suggests 3 or more servings of milk contribute to increases in BMI in adolescents as a result of excess energy intake [27]. Heaney and colleagues suggest increasing the intake of 1 serving of dairy at equivalent energy intake decreases a gain of a 1–2 kg in young children. Most of the studies in children/adolescents are modest in sample size and none have examined the issue of calcium or dairy products and weight in African-Americans.

Increasing dairy intake is intermingled with total energy intake. Some but not all studies show that by increasing dairy intake is compensated by the decline in energy from all foods [13]. Estimating total energy intake in free living adolescents is difficult at best. The average energy intake reported by our sample was lower than what would be expected using the equations to determine energy requirement for overweight males and females [21]. Using the ratio of reported energy intake to estimated intake using EER [28], we found that 95% of the sample underreported their intake. Misreporting did not affect the number that met the RDI for any of the calcium groups. However, the mean reported energy intake to EER ratio was similar (0.59–0.81) to that reported for US children and adolescents between age 12 and 18 years [29]. Whether the differences between actual and estimated intake were due to underreporting or under-eating during the recording period cannot be determined from our data. 

Lower reported energy intake may or may not translate into a lower quality of diet depending on the selection of food. When high-calcium foods are consumed by children and adolescents in the US, the trend is toward choosing high-fat cheese and ice cream instead of low-fat dairy products [30]. In our sample, the percent meeting the ADMR for fat (25–35%) increased with increasing calcium intake, suggesting that they may have selected sources of dairy that are lower in fat. Studies show that AA can include dairy products in their diet without symptoms of lactose maldigestion, by including milk in mixed meals, using lactose enzyme aids, or consuming low lactose products such as cheese or yogurt [31]. Our data shows that those in the highest quartile of dairy intake consumed dairy containing lactose without incident. Those in the highest quartile of calcium intake had higher intake of potassium, vitamin A and fiber, suggesting a higher intake of fruits and vegetables. Like other reports [32,33,34,35], our data support a view that the quality of the diet improves with higher calcium intake from milk products. Dairy products provide important sources of phosphorus, magnesium, riboflavin and potassium [33,36,37,38] that aid in meeting the DRI for these nutrients.

Weight status using the Centers for Disease Control classification documents in the US 22 percent of AA youth between age 12 and 19 years had a BMI >95th percentile for age and gender, and 38.1% had a BMI above the 85th percentile [3]. Given the grave implications for metabolic disorders, an expert committee on prevention, assessment and treatment of obesity in children and adolescents has suggested that a weight classification (severe obesity) be added for those >99th percentile for age and gender [39] The severe obesity translates into a BMI between 30 to 32 kg/m^2^ for youths 10 to 12 years of age and BMI >34 kg/m^2^ for youths 14 to 16 years of age. Using this definition, 63% of our population would be characterized as extremely obese. 

Eighty four percent of our sample had one or more metabolic risk factors for cardiovascular disease. This prevalence is in agreement with previous reports in children/adolescents [14,37,40,41,42]. Pereira *et al.* [43] recently found that among young, overweight adults in the CARDIA study, dairy consumption (100% dairy foods plus foods with dairy as the main ingredient) was inversely associated with the incidence of all components of the metabolic syndrome over 10 years, independent of ethnicity, gender, other lifestyle factors, and macronutrient and micronutrient intakes. However, incongruence with others [17] we found no association between daily dairy intake and the presence of dyslipidemia, hypertension, severity of overweight, or abnormal glucose metabolism. 

Children and adolescents at risk for overweight and obesity have a higher risk of becoming overweight or obese adults [44]. A question remains as to whether a high-dairy would attenuate our participants’ comorbid conditions. In adults, increasing calcium intake during weight loss improves lipids in adults [45]. Although dairy intake has been associated glucose metabolism and insulin resistance [43], the role of calcium in controlling blood pressure in adults varies from study to study [46]. Investigations regarding the association of calcium intake and comorbidities raise the question of whether the differences in calcium and vitamin D metabolism found between AA and Caucasians with regard to the skeletal system [47,48,49] are related to the racial differences seen in the prevalence of obesity, glucose intolerance or type 2 diabetes [43,50,51,52] The efficacy of dairy intake on weight loss or change in metabolic indices in children and adolescents is an opportunity for further research efforts.

The strength of our report lies in uniqueness of our sample children. A limitation of our report is that few of our participants reported food intake that met the DRI for calcium. With a larger distribution of calcium intake we might have seen an association using calcium as a continuous variable. Using a sensitivity analysis based on quartiles of calcium intake specific for females, our data show that a diet with very low calcium intake (<314 mg/day) appears to affect the level of fat mass. Whether this association is spurious due to chance or is real requires confirmation in other cohorts similar to ours. Given that our population is characterized by extremely overweight AA’s, a sample that has a wider BMI range may yield different results. A limited range of body fat and dietary calcium intake may have made it difficult to examine relationships between nutrients and body weight status. The cross-sectional nature of the investigation reflects a point in time and cannot be projected as long-term risk nor support causation.

## 4. Conclusions

This study clearly documented that 84% of extremely overweight AA children and adolescents have metabolic disorders that increase risk of cardiovascular disease. Those girls with the lowest intake of calcium had increased body fat and lower mineral and vitamin intake compared to those with a highest intake of calcium. Twenty-five percent of the study population consumed lactose-containing dairy products without adverse effects, suggesting that an intervention focused on increasing calcium intake from dairy may increase key nutrients and perhaps be beneficial for body composition in AA girls.
